# A Clickable Analogue of Ketamine Retains NMDA Receptor Activity, Psychoactivity, and Accumulates in Neurons

**DOI:** 10.1038/srep38808

**Published:** 2016-12-16

**Authors:** Christine Emnett, Hairong Li, Xiaoping Jiang, Ann Benz, Joseph Boggiano, Sara Conyers, David F. Wozniak, Charles F. Zorumski, David E. Reichert, Steven Mennerick

**Affiliations:** 1 Department of Psychiatry, Washington University School of Medicine, 660 S. Euclid Ave., St. Louis, MO 63110, USA; 2 Graduate Program in Neurosciences, Division of Biology and Biomedical Sciences, Washington University School of Medicine, 660 S. Euclid Ave., St. Louis, MO 63110, USA; 3 Department of Radiology, Washington University School of Medicine, 660 S. Euclid Ave., St. Louis, MO 63110, USA; 4 Taylor Family Institute for Innovative Psychiatric Research, Washington University School of Medicine, 660 S. Euclid Ave., St. Louis, MO 63110, USA; 5 Department of Neuroscience, Washington University School of Medicine, 660 S. Euclid Ave., St. Louis, MO 63110, USA.

## Abstract

Ketamine is a psychotomimetic and antidepressant drug. Although antagonism of cell-surface NMDA receptors (NMDARs) may trigger ketamine’s psychoactive effects, ketamine or its major metabolite norketamine could act intracellularly to produce some behavioral effects. To explore the viability of this latter hypothesis, we examined intracellular accumulation of novel visualizable analogues of ketamine/norketamine. We introduced an alkyne “click” handle into norketamine (alkyne-norketamine, A-NK) at the key nitrogen atom. Ketamine, norketamine, and A-NK, but not A-NK-amide, showed acute and persisting psychoactive effects in mice. This psychoactivity profile paralleled activity of the compounds as NMDAR channel blockers; A-NK-amide was inactive at NMDARs, and norketamine and A-NK were active but ~4-fold less potent than ketamine. We incubated rat hippocampal cells with 10 μM A-NK or A-NK-amide then performed Cu^2+^ catalyzed cycloaddition of azide-Alexa Fluor 488, which covalently attaches the fluorophore to the alkyne moiety in the compounds. Fluorescent imaging revealed intracellular localization of A-NK but weak A-NK-amide labeling. Accumulation was not dependent on membrane potential, NMDAR expression, or NMDAR activity. Overall, the approach revealed a correlation among NMDAR activity, intracellular accumulation/retention, and behavioral effects. Thus, we advance first generation chemical biology tools to aid in the identification of ketamine targets.

The non-competitive N-methyl-D-aspartate receptor (NMDAR) antagonist, ketamine, is a psychotomimetic, dissociative anesthetic, and fast acting antidepressant in humans^1^,^2^,^3^,^4^,^5^ and has antidepressant-like actions in rodents^6^,^7^,^8^. Many questions remain about cellular effects underlying these actions. For instance, drugs with very similar actions on NMDARs may not share ketamine’s behavioral effects^9^. One explanation is that metabolites of ketamine could have psychoactive effects^10^. Another non-exclusive possibility is that ketamine or its metabolites could have undiscovered cellular targets relevant to their behavioral effects. For instance, as a weak base, the neutral species of ketamine may readily permeate cell membranes and bind intracellular targets^11^,^12^,^13^. Targets other than NMDARs have recently been proposed to underlie antidepressant effects^10^. However, the nature and mechanisms of any intracellular accumulation of ketamine are unclear.

Ketamine is an open channel blocker of NMDARs, a major class of glutamate receptors governing excitation in the vertebrate CNS. It is unusual among psychoactive NMDAR channel blockers in yielding a major metabolite, norketamine, which is also psychoactive. Although its plasma concentrations remain low relative to peak ketamine levels, norketamine has a significantly longer half-life in humans than ketamine (11 h vs. 2.5 h)^14^,^15^. Norketamine blocks NMDARs, but its detailed pharmacodynamic properties have not been explored^16^. It is also unclear whether norketamine possesses the acute psychoactive and antidepressant activity of the parent compound.

Given that targets other than NMDARs may be important for some behavioral effects of ketamine, cellular visualization of ketamine analogues could help reveal unanticipated targets relevant to drug effects. Here, we use “click” chemistry, an approach that allows visualization of biologically active molecules by covalently linking them to their substrates and/or visual probes, to probe the possibility of intracellular accumulation.

To address competing ideas regarding ketamine’s actions, we tested ketamine, norketamine, and two novel analogues that can be visualized with click chemistry: alkyne norketamine (A-NK) and alkyne norketamine amide (A-NK-amide), a non-protonatable analogue. We found that three of the four compounds exhibited strong psychoactive effects, including antidepressant-like effects in the rodent forced swim test (FST). The fourth compound, A-NK-amide, was much weaker in behavioral assays. The three behaviorally active compounds exhibited channel block at NMDARs in hippocampal neurons, while A-NK-amide exhibited much weaker activity at NMDARs. A-NK-amide, unlike the active analogue A-NK, failed to strongly accumulate in intracellular compartments, retrospectively assessed using *in situ* click chemistry on dissociated, fixed neurons. The subcellular compartments labeled by A-NK were diverse, suggesting the possibility of multiple intracellular targets correlated with psychoactive effects. Thus, intracellular accumulation correlated with cellular and behavioral effects.

Our work establishes that the major metabolite of ketamine has psychoactive and antidepressant-like effects and demonstrates the importance of protonation for cell entry and/or retention. We introduce for the first time a visualizable probe that retains electrophysiological and behavioral properties of ketamine/norketamine while enabling visualization of drug localization. The probe revealed intracellular labeling potentially relevant to cellular actions of ketamine/norketamine.

## Results

### Norketamine and A-NK exhibit strong psychoactivity; A-NK-amide is weaker

**Figure 1:**
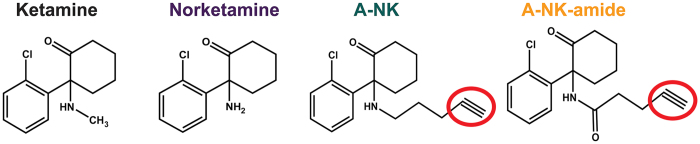
Structures of compounds used in the studies. The red ovals highlight the alkyne group exploited for click chemistry visualization. Colors of names are retained throughout figures in symbols, traces, and bar fills for the indicated compounds.

The compounds evaluated in the present work are shown in Fig. 1. Recent structure-activity work on ketamine showed that the methyl group on the central nitrogen atom is expendable for anesthetic activity^17^, so we performed chemical modifications with norketamine as precursor to the chemical biology analogues A-NK and A-NK-amide.

Ketamine exhibits a range of psychoactive effects, from acute (e.g., psychotomimetic, anesthetic at high doses) to delayed (e.g., antidepressant at low doses) behavioral changes. We first examined near-anesthetic doses of compounds to test acute psychoactive effects^18^. We quantified locomotor activity and rearing behavior for 60 min following a single i.p. injection. Ketamine at 100 mg/kg (n = 8) significantly increased the number of ambulations across the 60-min test session relative to vehicle controls (n = 7)[Treatment effect: F(1,13) = 11.88, p = 0.004], and the differences varied with time [Treatment x Time Blocks interaction: F(5,65) = 5.26, p = 0.001] (not shown). Further, ketamine decreased behavioral rearing frequency [Treatment effect: F(1,13) = 46.47, p < 0.00005], which also varied with time [Treatment x Time Blocks interaction: F(5,65) = 8.16, p = 0.0002] (not shown). Subsequent pair-wise comparisons confirmed that ketamine reduced rearing beyond Bonferroni multiple comparisons correction (p < 0.005) across the first 50 min of the test session. Differences were significant for ambulations during only the first 20 min (blocks 1, 2; p < 0.003). These results confirm observations on ambulatory activity and rearing by others^18^.

**Figure 2:**
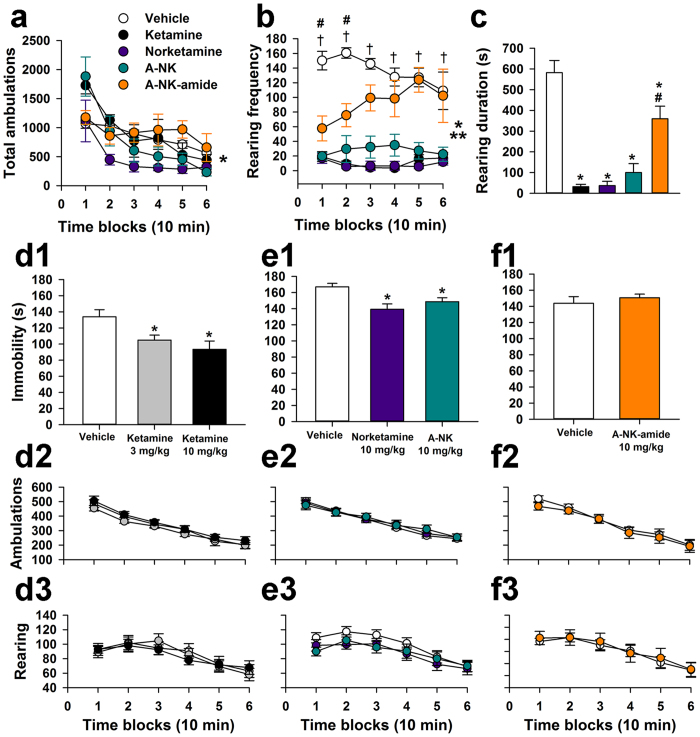
Ketamine, norketamine and A-NK, but not A-NK-amide, exhibit strong psychoactivity. (**a**) Ambulatory activity following i.p. drug injection at 50 mg/kg (ketamine) or 100 mg/kg (other compounds). rmANOVA showed a significant Treatment x Time Blocks interaction, [F(20,100) = 2.57, *p = 0.004], but additional contrasts showed no consistent drug induced change relative to vehicle controls (n = 5 animals per condition). (**b)** All drug treatments decreased vertical rearing frequency [Treatment effect: F(4,20) = 30.10, **p < 0.00005; Treatment x Time Blocks interaction: F(20,100) = 2.42, *p = 0.009], although the effect was smaller and more short-lived for A-NK-amide. Specifically, rearing was reduced (beyond Bonferroni correction) in the ketamine, norketamine, and A-NK groups for all time blocks (^†^p < 0.008), but A-NK-amide reduced rearing for only the first two time blocks (^#^p < 0.008). (**c**) Ketamine and analogues also decreased average rearing duration in the same test session [Treatment effect: F(4,20) = 30.51, p < 0.00005]. Durations were significantly decreased in each of the drug treatment groups relative to vehicle controls (*p < 0.002). However, rearing duration in the A-NK-amide group was significantly greater than duration in the other drug groups (^#^p < 0.0005). For A-C, n = 5 mice per condition. (**d1**) Ketamine reduced immobility time measured during the FST in mice 3 hours after administration of the indicated concentrations of ketamine (Treatment effect: p = 0.005). Both 3 mg/kg and 10 mg/kg significantly reduced immobility time (*p = 0.021 and 0.002, respectively, n = 20 mice per condition). (**d2**,**d3**) In separate experiments using control animals from the FST test, ambulatory activity and rearing behavior were evaluated 3 hr following following drug injection (Symbol colors as in D1, n = 13 mice per condition). No effect of drug on locomotion or rearing was detected. (**e1–e3**) Norketamine and A-NK (10 mg/kg) also significantly reduced immobility time in the FST (Treatment effect: p = 0.002; *p = 0.0005 and 0.019, respectively; n = 32–34 mice per condition), but had no effect on ambulations or rearing (n = 17–27). (**f1–f3**) A-NK-amide (10 mg/kg) did not significantly alter immobility time compared with cohort-matched controls (n = 25 per condition). General ambulatory activity and rearing were unaffected (n = 12 per condition). In D1 and E1 asterisks indicate significantly different from vehicle control in pairwise comparisons following ANOVA.

Based on these results, we focused on rearing behavior as a sensitive indicator of psychoactive effects of ketamine-like compounds in the C57Bl/6 J mice studied here. In an interleaved cohort of animals, ambulatory activity following ketamine (50 mg/kg), norketamine, A-NK, and A-NK-amide (100 mg/kg each) did not differ among drugs or against vehicle control (Fig. 2a; n = 5 for each group), suggesting no anesthetic effects of any of the drugs. Doses were chosen based on relatively potencies from previous work^16^. There was, however, a drug by post-injection time interaction effect on ambulation (Fig. 2a), hinting at differences in psychoactivity sometime during the test session. In the more sensitive measure of rearing frequency, ketamine, norketamine, and A-NK had strong effects (Fig. 2b), but A-NK-amide was weaker than the other compounds; rearing frequency was significantly reduced in the A-NK-amide treated mice only early in the session (Fig. 2b). Rearing duration showed similar effects (Fig. 2c). Collectively these results indicate that high, subanesthetic doses of ketamine, norketamine, and A-NK robustly depress rearing behavior, while A-NK-amide induces weaker psychoactive effects compared with ketamine and the other two analogues.

To test effects in the FST, a screen for antidepressant drugs, mice were dosed i.p. with 3 or 10 mg/kg of ketamine or with vehicle (n = 20 per condition) and then tested 3 h later on the FST^6^,^8^,^9^,^19^. An ANOVA conducted on these data (Fig. 2d1) revealed a significant effect of Treatment, [F(2,57) = 5.89, p = 0.005] on immobility time, with subsequent pair-wise comparisons showing that both the 3 and 10 mg/kg doses significantly reduced immobility, (p = 0.021 and 0.002, respectively) compared to vehicle-treated mice. Another independent cohort of naive mice was injected with 3 or 10 mg/kg of norketamine or vehicle and then assessed on the FST to test the hypothesis that the primary metabolite of ketamine (i.e., norketamine), may also have antidepressant-like properties. An ANOVA of these data yielded a significant effect of Treatment, [F(2,46) = 5.31, p = 0.008], on immobility time (n = 16–17 mice per condition). Follow-up comparisons indicated that the 10 mg/kg dose of norketamine resulted in a significant (beyond Bonferroni correction: p = 0.025) reduction in immobility time (p = 0.003) while the 3 mg/kg dose also decreased immobility (p = 0.039), relative to vehicle controls (data not shown). We replicated the norketamine (10 mg/kg) finding alongside a test of A-NK (10 mg/kg), a synthetic analogue of norketamine in a separate cohort of animals (Fig. 2e1). An ANOVA of these data (n = 32–34 mice per condition) indicated a significant Treatment effect, [F(2,97) = 6.73, p = 0.002], on immobility and pair-wise comparisons showed that both norketamine and A-NK significantly reduced immobility relative to vehicle control injections (p = 0.0005 and 0.019, respectively). In contrast, injection of the other synthetic analogue, A-NK-amide, failed to affect immobility time in the FST compared to vehicle control injections (Fig. 2f1) in additional independent groups of naive mice (n = 25 per condition).

The FST was tested with a 3 hr delay and lower dose compared with initial tests of acute psychoactivity (Fig. 2a–c), so acute psychotomimetic or anesthetic effects should not have affected performance during the FST. Nevertheless, we examined the effects of ketamine, norketamine, A-NK, and A-NK-amide on general ambulatory activity and vertical rearing using the same dosing and post-injection delay used for FST testing. Ketamine (3 mg/kg and 10 mg/kg; n = 13 per condition) failed to show significant effects of either dose on ambulatory activity (total ambulations), and the same was true for norketamine (n = 17), A-NK (n = 18), and A-NK-amide (10 mg/kg, n = 12; Fig. 2d2). In addition, none of the drug treatments had any significant effects on vertical rearing frequency at this later time point and lower dose (Fig. 2d3,e3,f3). These data suggest that it is unlikely that ketamine, norketamine or A-NK altered motor activity to cloud interpretation of the antidepressant-like effects of the drugs during the FST.

### Voltage dependence and kinetic studies of analogues

**Figure 3:**
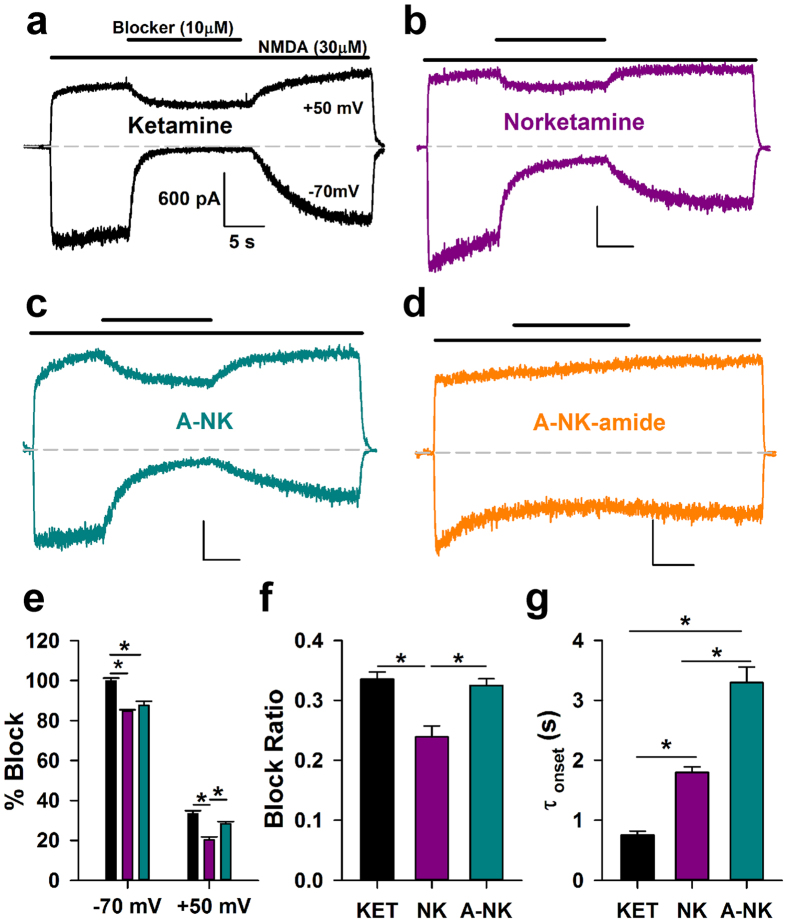
Ketamine, norketamine and A-NK are NMDAR antagonists. (**a–d)** Representative traces depicting block and wash off at −70 mV and +50 mV for 10 μM ketamine (**a**), 10 μM norketamine (**b**), 10 μM A-NK (**c**), and 10 μM A-NK-amide (**d**). Calibration bar values in (**a**) correspond to all 4 panels. All the compounds, save A-NK-amide, were active. (**e)** Inhibition achieved near steady-state for the active compounds was calculated. At −70 mV and at +50 mV, both norketamine and A-NK were significantly different than ketamine, but at +50 mV, norketamine exhibited significantly less block than the two compounds (n = 4–5, *p < 0.05, t-test Bonferroni correction for multiple comparisons). (**f)** The inhibition at +50 mV was divided into the block achieved at −70 mV to calculate a “block ratio” for all active compounds. This measure of voltage-sensitivity was significantly lower for norketamine compared to the two other compounds (n = 4–5 *p < 0.05, t-test Bonferroni correction for multiple comparisons). (**g**) The rate of block onset at −70 mV was measured for all three active compounds. Norketamine exhibited significantly slower onset kinetics than ketamine, and A-NK displayed slower kinetics than both ketamine and norketamine (n = 4–5, *p < 0.05, t-test with Bonferroni correction for multiple comparisons).

There have been few evaluations of the effect of norketamine on NMDAR function^16^,^20^. No work has assessed activity of chemical biology analogues of ketamine/norketamine at NMDARs. In dissociated cultures of rat hippocampal neurons, we explored properties of the analogues on NMDAR function. All three NMDAR-active compounds exhibited voltage dependence at equimolar concentration (10 μM; Fig. 3), suggesting similar mechanism of action. Norketamine and A-NK exhibited indistinguishable block at −70 mV but significantly different block at +50 mV, suggesting that A-NK exhibits slightly weaker voltage dependence (Fig. 3e,f). Interestingly, the amide derivative was completely inert at 10 μM (Fig. 3d). At −70 mV both norketamine and A-NK displayed significantly slower onset kinetics than ketamine, with A-NK exhibiting especially slow onset and offset kinetics during continuous agonist exposure (Fig. 3g). In summary, both norketamine and A-NK retained voltage dependence similar to ketamine, with subtle differences in kinetics, suggesting that both mimic the actions of the parent compound.

**Figure 4:**
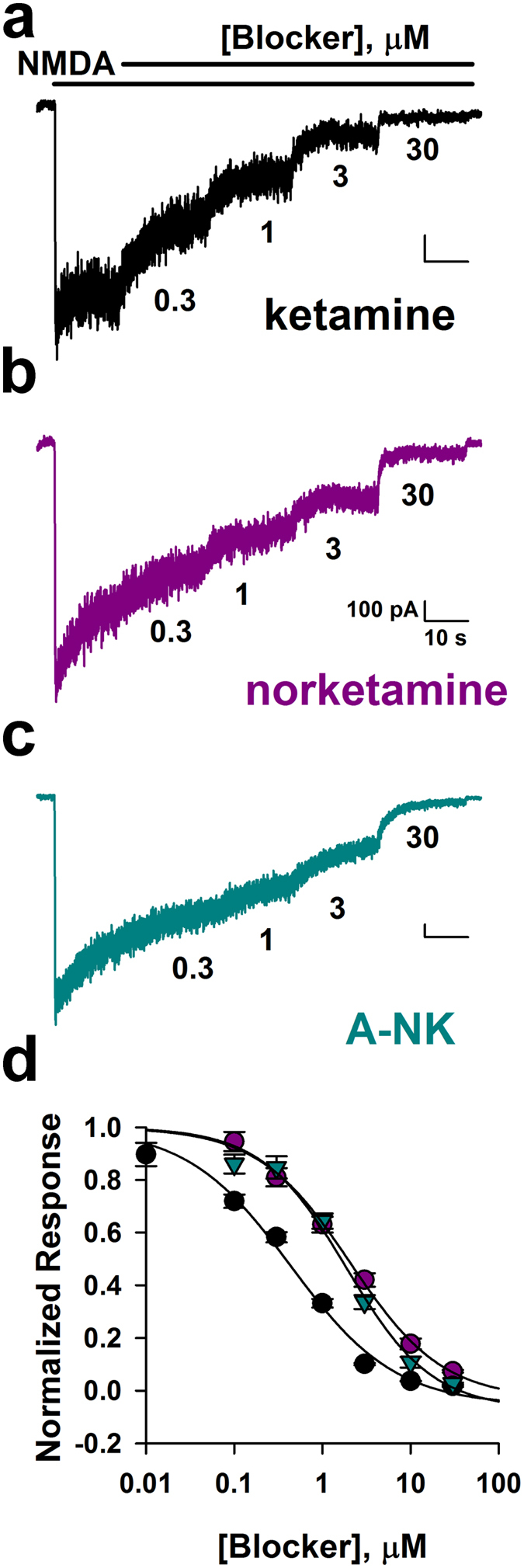
Norketamine and A-NK are less potent than ketamine. (**a–c)** Representative traces depict inhibition by increasing concentrations (0.3, 1, 3, 30 μM) of ketamine (**a**), norketamine (**b**) and A-NK (**c**). Concentrations are given below each trace. (**d)** Steady-state block achieved in pooled results from cells challenged with the indicated concentrations. Concentration-response curves for ketamine, norketamine, and A-NK are shown (n = 9–10). Solid lines represent fits to the Hill equation of the form I = I_max_* C^*n*^/(IC_50_^*n*^ + C^*n*^), where I_max_ is maximum current in the absence of agonist, C is antagonist concentration, *n* is the Hill coefficient, and IC_5α_s the concentration inhibiting 50% of the response. The respective IC_50_ values and Hill coefficients for the compounds were estimated from fits to be: 0.4 μM and 0.7 (ketamine), 2.0 μM and 0.8 (norketamine) and 1.8 μM and 0.9 (A-NK). Ketamine was significantly more potent than norketamine and A-NK (p < 0.05, unpaired t-tests, from EC_50_ values obtained from fits to individual cells).

To compare relative potencies of the active analogues, we challenged cells with increasing concentrations of antagonist in the prolonged presence of 10 μM NMDA as agonist (Fig. 4). We found that norketamine was approximately 4-fold less potent than ketamine (IC_50_ ketamine: 0.4 μM, IC_50_ norketamine: 2.0 μM, n = 9–10, Fig. 4a,b,d) and A-NK was equipotent to norketamine (IC_50_: 1.8 μM, n = 9, Fig. 4c,d).

**Figure 5:**
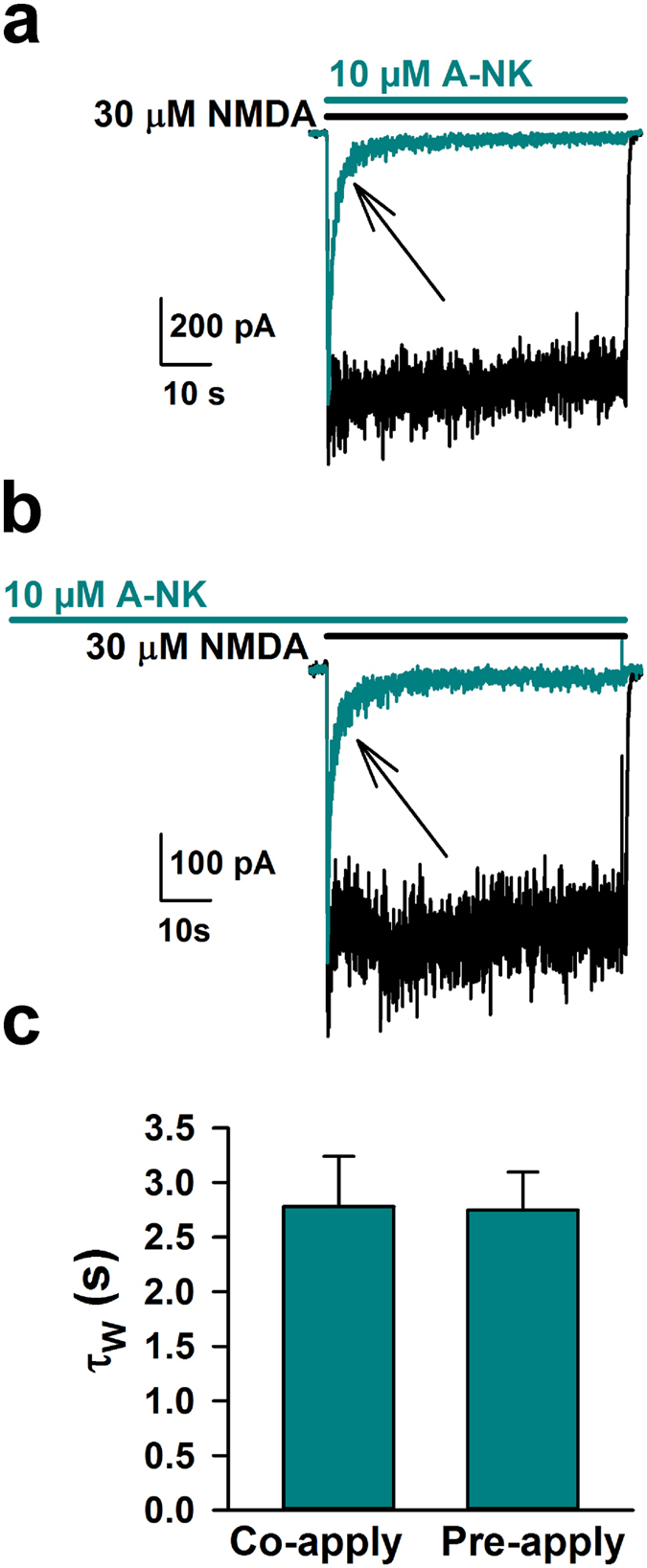
Pre-application of A-NK does not speed rate of block. (**a)** Representative trace of control current (black) in response to 30 μM NMDA or of current following a saline pre-application and immediate co-application of NMDA and 10 μM A-NK. (**b**) Same as in A, except prior to the cyan trace, A-NK was pre-applied for 60 seconds. (**c**) The rate of onset of block (depicted by arrows in **a**, **b**) was calculated for both protocols by fitting a bi-exponential function to the drug onset and calculating a weighted time constant (τ_w_). There was no significant difference between blocking rates during the different pre-application protocols (n = 5 each, p > 0.05, unpaired t-test).

Figures 3 and 4 suggest that A-NK has slower kinetics than the parent compounds when applied during continuous agonist presentation. The most direct comparison is between A-NK and norketamine, because these two compounds proved equipotent at −70 mV. We and others have previously shown that kinetics of trapping NMDAR channel blockers, including ketamine, is rate limited by low channel open probability^21^,^22^. The additional slowness of A-NK could result from intrinsically slow pharmacodynamics (drug binding and dissociation), or it could represent slow access to the NMDAR through routes other than aqueous diffusion. For instance, previous work has shown that ketamine can reach at least one site via a lipophilic pathway^23^. Given the enhanced lipophilicity that the hydrocarbon chain imparts to the analogue (cLogP A-NK: 3.86 ± 0.54 vs. ketamine: 2.75 ± 0.33 vs. norketamine: 2.32 ± 0.33, see Methods), we hypothesized that A-NK may need to reach a *non-aqueous* binding site, causing kinetics to be rate limited by membrane partitioning, as seen with steroid modulators of GABA_A_ receptors^24^. This is in contrast to local anesthetics acting at voltage-gated sodium channels, where hydrophobicity and a membranous access route speed drug actions^25^,^26^. If cellular accumulation or compartmentalization explains the slow kinetics of A-NK, then A-NK pre-application should speed the block observed upon ensuing agonist/A-NK co-application. To test this, we pre-applied A-NK for 60 s, followed by co-application with agonist. We compared kinetics of block onset with kinetics observed in a co-application-only protocol. The rate of A-NK block upon NMDA co-application was unaffected by A-NK pre-application (Fig. 5). This suggests that A-NK pharmacodynamics accounts for the slow block during continuous agonist presentation, perhaps because of steric-hindrance from the bulky side chain. These data also support the idea that A-NK, like ketamine, is a use-dependent blocker because in the pre-application protocol, there was no evidence that A-NK interacted with the receptor/channel before agonist was presented^27^.

**Figure 6:**
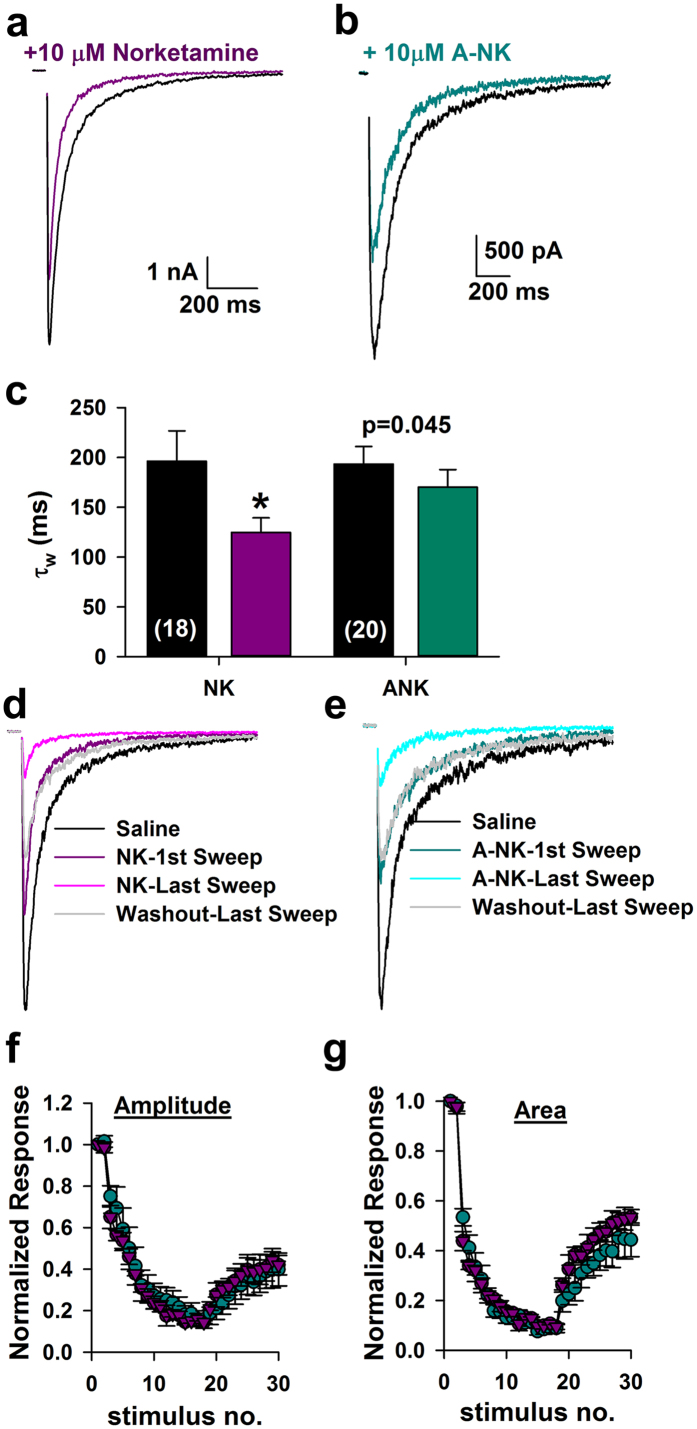
A-NK inhibits synaptic EPSCs similarly to norketamine. (**a,b**) Representative traces of autaptic NMDAR EPSCs in saline (black) and the initial sweep following pre-application with 10 μM norketamine (**a**) or 10 μM A-NK (**b**). Norketamine and A-NK were applied to separate cells with representative traces shown from the two populations. (**c)** A weighted tau (τ_w_) was calculated from a bi-exponential fit to the decay of the EPSC. Norketamine significantly accelerated the rate of decay compared to saline controls (n = 18, *p < 0.05, paired t-test). A-NK reduced EPSC decay τ_w_ at trend level (p = 0.045, n = 20, paired t-test). (**d,e)** Representative traces from different cells in which progressive block by the compounds was assessed. Legends indicate the identity of the colored traces. (**f,g)** Plots of peak and total postsynaptic charge, normalized to baseline EPSCs before drug application, demonstrating that norketamine and A-NK reached maximum block and were washed out with an indistinguishable time course (n = 6–7, p > 0.05 unpaired t-test). Intersweep interval was 30 seconds in all recordings.

Results from Figs 3, 4 and 5 show that the psychoactive compound A-NK inhibits responses to sustained agonist application. However, does the slow kinetics of A-NK hinder its ability to depress synaptic responses, which are generated in response to very brief agonist presentation? To address this question we applied norketamine and A-NK prior to evoking NMDAR-mediated EPSCs. Both drugs significantly reduced the peak of the EPSC (norketamine: 22.6 +/− 3.0% reduction from baseline, n = 18, p < 0.05 paired t-test; A-NK: 30.5 ± 2.9% reduction from baseline, n = 20, p < 0.05). A-NK’s effect on the EPSC decay time course was only trend level (p = 0.045) compared with a stronger effect of norketamine (Fig. 6a–c). Nevertheless, upon repetitive stimulation at 0.033 Hz, both norketamine and A-NK behaved nearly indistinguishably (Fig. 6d–g). Both had strong, cumulative effects on EPSC peak and total charge that slowly returned toward baseline following compound removal (Fig. 6f,g). These results are consistent with the idea that the primary mode of norketamine and A-NK inhibition of NMDA receptor function is through activation-dependent trapping block, similar to ketamine^28^,^29^,^30^. We conclude that the slower kinetics of A-NK do not prevent it from interacting with synaptic neurotransmission.

### A-NK intracellular accumulation

**Figure 7:**
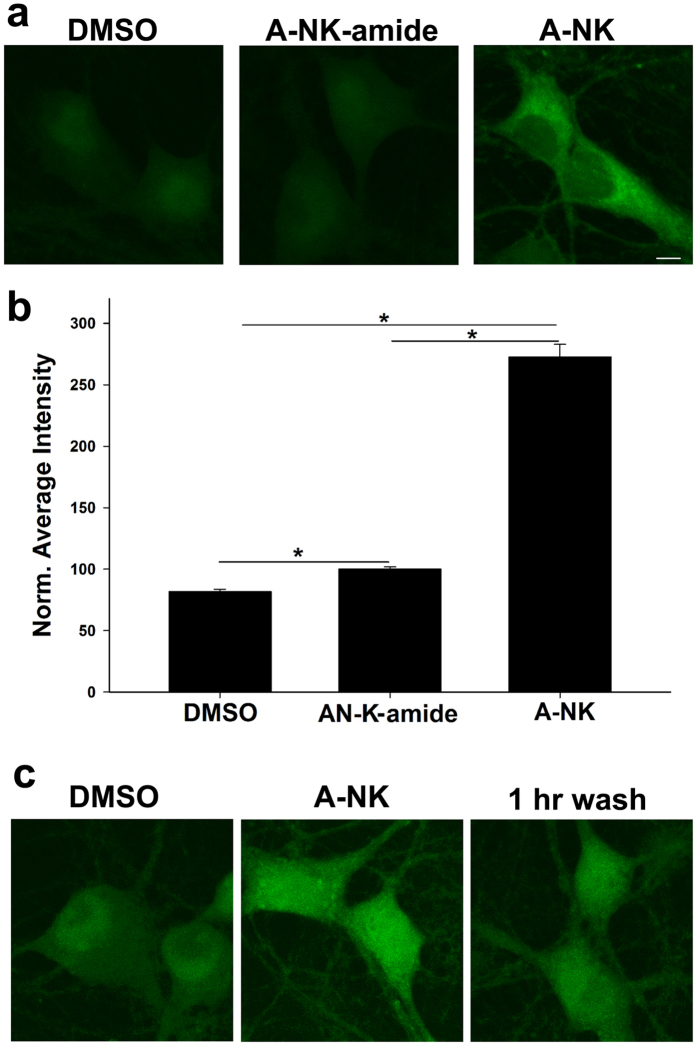
A-NK but not A-NK-amide strongly accumulates inside neurons. (**a**) Confocal imaging depicting cells exposed to DMSO, 20 μM A-NK-amide or 20 μM A-NK, permeabilized and stained with azide-Alexa Fluor 488 under copper-catalyzed “click” reaction conditions. Labeling is observed in the A-NK treated dish but is much weaker for A-NK-amide. Scale bar: 5 μm. (**b**) The amount of fluorescence in each condition was quantified (n = 15 neurons from 3 separate experiments, *p < 0.05, paired t-test). (**c)** Cells were incubated in 10 μM A-NK for 1 h, followed by fixation. Cells were either processed immediately for click cyto-fluorescence or were washed for 1 h in phosphate-buffered saline before processing. Images are representative of three independent experiments. Scale bars in A and C: 5 μm.

One recent hypothesis suggests that ketamine may trigger antidepressant downstream signaling pathways by binding nascent intracellular NMDA receptors resident in the endoplasmic reticulum^12^. A-NK and ANK-amide allowed us to incubate live cells with the unlabeled analogue, then visualize the localization of analogue following cell fixation and *in situ* fluorescence click chemistry with a fluorescent dye. After bath incubation with unlabeled A-NK or A-NK-amide in live cells, we washed away extracellular analogue, fixed cells and “clicked” azide-Alexa Fluor 488 to residual compound (see Methods). Because localization occurred in fixed cells when excess extracellular alkyne was no longer present, we posit that this protocol should reveal sites of localization of the alkyne analogues at the time of fixation. We observed intracellular labeling in neurons, with accumulation of A-NK most evident. A-NK-amide accumulation was barely detectable above background (though statistically significant, Fig. 7a,b). Prolonged wash following fixation only modestly altered intensity and pattern of A-NK accumulation, suggesting that the analogue was indeed fixed in place by the protocol (Fig. 7c; n = 40 cells in 4 experiments, 64 ± 3% vs. 52 ± 3% increase in fluorescence with a 1 h wash following fixation, vs. DMSO control).

**Figure 8:**
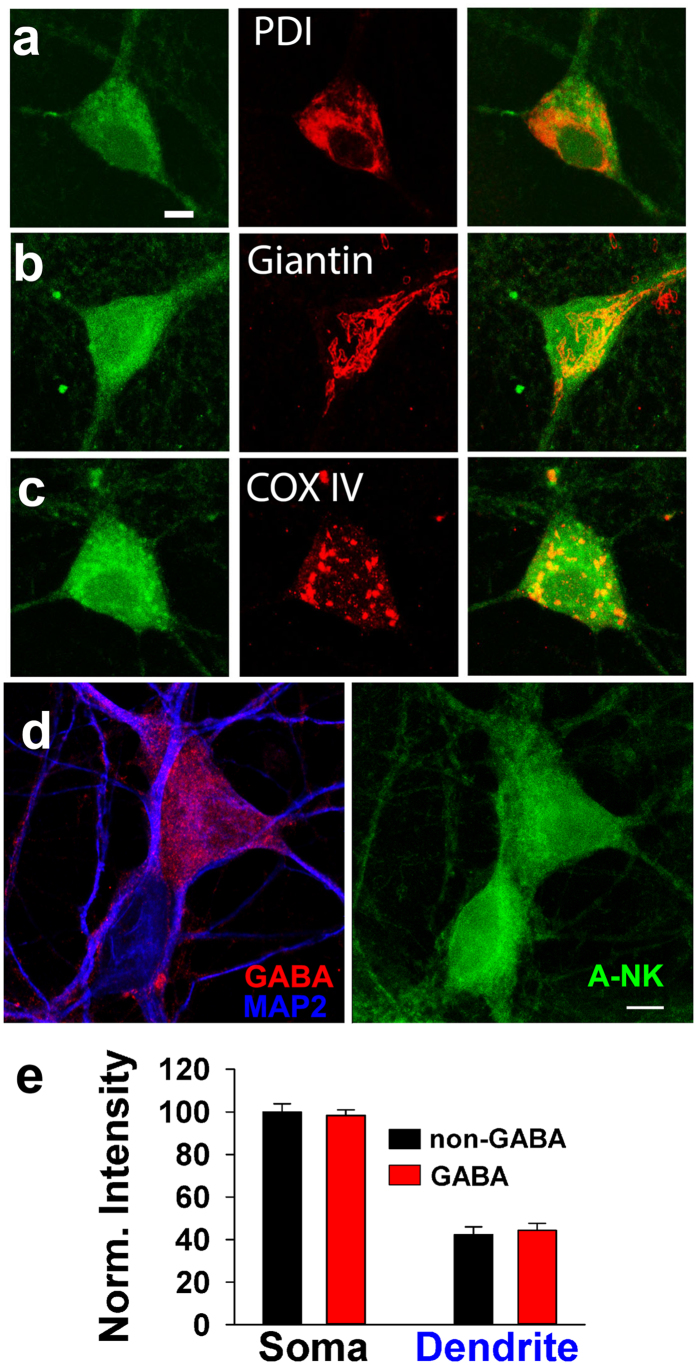
A-NK staining involves multiple intracellular compartments. (**a–c)** Left panels depict confocal imaging of staining of cells exposed to 10 μM A-NK, permeablized and stained with azide-Alexa Fluor 488 under copper-catalyzed “click” reaction conditions. Middle panels show immunofluorescence staining of the ER (anti-PDI, **a**), Golgi (anti-Giantin, **b**) or mitochondria (anti-COXIV, **c**). Right panels are merged images indicating that the A-NK staining did not overlay specifically with any of the organelle markers. Scale bar: 5 μm. (**d)** To test for preferential A-NK accumulation in dendrites and interneurons, we co-labeled with antibodies for GABA (red) and MAP2 (blue). The right panel shows A-NK accumulation. (**e)** Summary of A-NK fluorescence from confocal projections in somas and dendrites of interneurons (n = 19) and principal neurons (n = 17).

We tested whether A-NK accumulated in a specific cellular compartment by co-labeling with antibodies against proteins resident in specific organelles. Antibody co-labeling of A-NK with anti-PDI, an endoplasmic reticulum marker, failed to reveal specific overlap (Fig. 8a). Although overlap appeared higher with the Golgi marker giantin and the mitochondrial marker COXIV (Fig. 8b,c), the co-labeling revealed no selective accumulation in any one compartment. We conclude that robust intracellular labeling characterizes an active ketamine/norketamine analogue, but there appears to be no selective or specific compartmentalization.

Some studies suggest that ketamine may preferentially inhibit interneurons^8^ and/or may alter postsynaptic (dendritic) glutamate signaling^6^,^8^. To test whether A-NK compartmentalization can help explain these observations, we co-labeled neurons with an antibody directed against GABA, a broad marker of hippocampal interneurons, and MAP2, a marker of dendrites (Fig. 8d, left). We examined A-NK accumulation on somas and dendrites of GABA and non-GABA cells (Fig. 8d, right) and found no evidence for preferential compartmentalization in dendrites or in interneurons (Fig. 8e).

**Figure 9:**
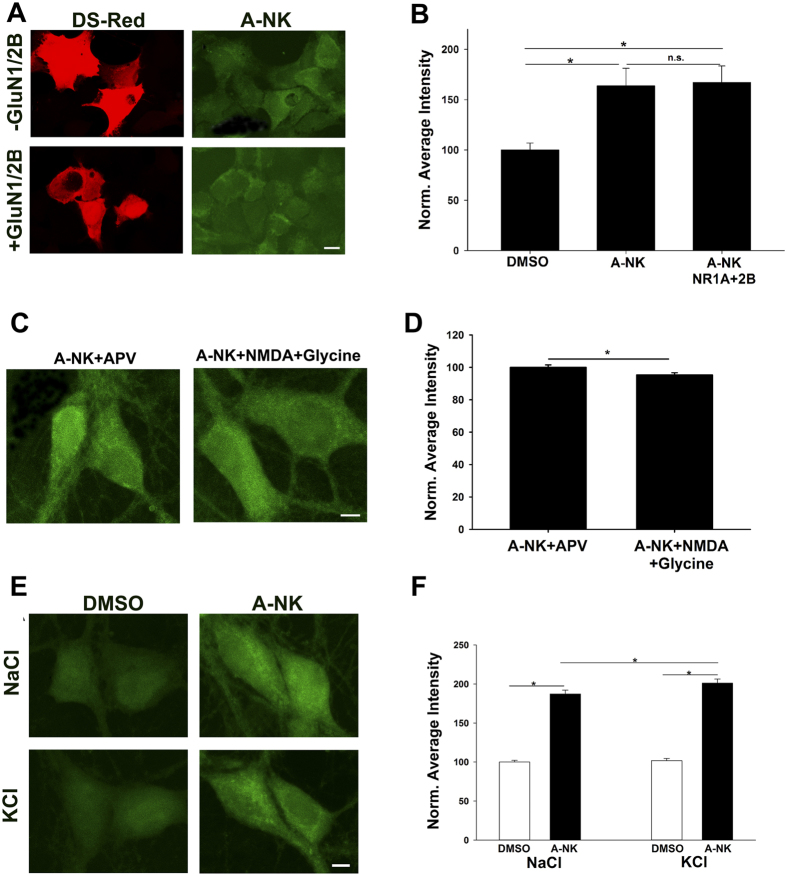
A-NK accumulation does not require NMDAR presence. (**a)** HEK cells were transfected with either DsRed alone or with NMDAR subunits GluN1a and GluN2B as indicated. Cells were incubated with 10 μM A-NK for 15 min, then fixed and processed for click cyto-fluorescence. Scale bar: 5 μm (**b)**. Quantification of fluorescence intensity from 5 independent experiments relative to sibling negative control dishes (DMSO treated; *p < 0.01). (**c,d)** Channel activation does not increase A-NK labeling in neurons. (**c)** Images of neurons co-incubated in 50 μM D-APV to prevent channel activation during A-NK exposure or in co-agonists 10 μM NMDA and 10 μM glycine to activate channels (1 h incubations). Scale bar: 5 μm. (**d)** Summary from 40 neurons in 4 independent experiments showing that channel activation slightly but significantly decreased A-NK accumulation (*p < 0.05), in contrast to expectations that permeation of the NMDAR may facilitate A-NK entry. (**e,f)** Depolarization does not reduce A-NK entry. (**e)** Neurons were incubated in 10 μM A-NK in normal saline or in saline containing 120 mM KCl to reduce the resting membrane potential to near 0 mV. Scale bar: 5 μm. (**f)** Summary of A-NK accumulation from 30 neurons in 3 experiments. Depolarization slightly increased A-NK accumulation (*p < 0.01), opposite of an electro-attraction mechanism of entry and retention.

To address whether intracellular accumulation is associated with nascent or recycled NMDARs, we used HEK cells, which do not express endogenous NMDARs. We transfected cells with DsRed and NMDAR subunits or DsRed alone as a control and imaged A-NK accumulation. We found that A-NK accumulated in both NMDAR-transfected cells and in control cells at similar levels (Fig. 9a,b), suggesting that intracellular accumulation is not accounted for by interaction with intracellular NMDARs.

This result also suggests that permeation of A-NK through surface NMDARs down its electrochemical gradient is not an important mechanism by which A-NK accumulates. To test this directly, we challenged neurons with A-NK while co-treating with 50 μM D-APV to prevent channel opening or while co-treating with agonist (10 μM NMDA and 10 μM glycine) to open channels and provide opportunity for A-NK entry. Subsequent visualization revealed that A-NK accumulation was not increased and in fact was slightly reduced by agonist co-treatment relative to D-APV treatment (Fig. 9c,d).

Finally, we addressed whether the difference in accumulation between positively charged A-NK and electroneutral A-NK-amide might result from electrostatic attraction of protonated A-NK to the negative membrane potential of the intracellular compartment. This experiment further tests the validity of the assumption that the uncharged form of ketamine is the only major permeant species. To test this, we incubated cells in A-NK with 120 mM KCl (replacement for NaCl) to reduce the membrane potential. If cell permeation was aided by negative membrane potential we would expect that intracellular staining would be reduced in cells treated with KCl. Instead, A-NK accumulation was slightly altered in the direction opposite of our prediction (Fig. 9e,f). Although we cannot fully exclude the possibility that membrane potential disruption caused by fixation influenced retention, the difference in A-NK and A-NK amide accumulation does not appear to be caused by electrostatic attraction.

## Discussion

Ketamine is a multifaceted psychoactive drug whose mechanisms of action are still under exploration. This paper addressed two questions regarding its actions. First, we examined the properties of norketamine, the major primary metabolite of ketamine, on psychoactivity, including performance during the FST, the most extensively used test for assessing the behavioral effects of antidepressant drugs in rodents^31^,^32^, and we also evaluated norketamine’s activity at NMDARs. Our work indicates that norketamine is subtly different than ketamine in its effects on NMDARs but could participate in the psychoactive actions of ketamine. Second, we introduce a novel chemical biology probe that has actions similar to ketamine and norketamine. Because of its similar effects, it and subsequent generations of analogues can be used to visualize and/or biochemically label novel targets of ketamine’s actions in neurons.

Previous work examining norketamine at NMDARs confirmed that it is neuroactive, but the work did little to characterize its mechanism of action. Here, we provide evidence that norketamine and the analogue A-NK both mimic ketamine’s actions at NMDARs and in tests of ketamine-like psychoactivity. Both norketamine and A-NK displayed characteristics consistent with voltage-dependent, open- channel blockers. The alkyne chain considerably slowed A-NK’s rate of block during exogenous agonist application (Figs 3 and 4). In contrast to steroid modulators of GABA_A_ receptors, for instance^33^, this slowing was not attributable to differences in the rate of access to the receptor. Instead, receptor interactions of A-NK are slow relative to norketamine, perhaps resulting from steric effects imparted by the alkyne side-chain. We note that all of our *in vitro* recordings were performed in the absence of physiological Mg^2+^, and Mg^2+^ influences the interaction of ketamine-like compounds with the NMDAR channel^9^,^34^. We therefore cannot exclude the possibility that Mg^2+^ might change the details of the various analogues’ interactions with NMDARs. In this regard, it is encouraging that our *in vivo* results (Fig. 1) largely parallel the *in vitro* findings.

Interestingly, we found that the A-NK-amide structure abolishes activity at NMDARs, has decreased behavioral effects, and reduces intracellular retention/labeling. We did detect significant short-term psychoactive effects (Fig. 2b,c) and significant intracellular fluorescence labeling above background levels, confirming the success of the click reaction (Fig. 7b). The weak A-NK-amide labeling could reflect poor permeation of the plasma membrane or poor intracellular retention. Either way, A-NK-amide seems to differ from the other analogues in its potential for interaction with intracellular targets. Permeation and retention could be governed by drug physicochemical properties. Estimated pKa values for the compounds in this study are as follows: ketamine (7.18), norketamine (6.78), A-NK (6.88), and A-NK-amide (−4.1). Calculated cLogP values are ketamine (2.75), norketamine (2.32), A-NK (3.86), and A-NK-amide (3.26). Thus A-NK-amide has high lipophilicity and is not protonated. It is therefore unclear what would reduce its permeation and retention; simple lipophilicity is often not sufficient to predict membrane permeability^35^. One possibility is that permeation of the positively charged analogue A-NK is facilitated by an active process that shuttles A-NK across the membrane. This process does not appear to involve permeation of the NMDAR channel itself or more general electro-attraction (Fig. 9). Another possibility is that by virtue of its status as a weak base, A-NK is better retained than A-NK-amide, perhaps in part by being trapped in its protonated state within acidic organelles^13^ and subsequently fixed in place. Binding to intracellular NMDARs does not appear to constitute a major mechanism of intracellular retention of A-NK (Fig. 9a,b).

Although A-NK exhibited more intracellular uptake/retention compared with A-NK-amide, A-NK did not exhibit clear selective partitioning into a single organelle sub-compartment within neurons. We consider two caveats to the conclusion that A-NK yields a realistic picture of ketamine/norketamine intracellular distribution. First, A-NK was not anchored before imaging, so diffusion beyond initial preferential sites of accumulation could have occurred during processing. Wash following fixation did not notably alter the accumulation pattern (Fig. 7c), suggesting that the compound is ultimately fixed in place, but we cannot exclude rapid redistribution during fixation. This issue has been dealt with in other contexts by introducing a photolabel to anchor the analogue *in situ* prior to fixation^36^,^37^. On the other hand, non-covalently anchored optical probes retain specific organelle distribution in other situations^38^,^39^,^40^. A second caveat is that the higher lipophilicity of A-NK over norketamine and ketamine may lead to a different distribution than the parent compounds. Because A-NK-amide, which shares high lipophilicity with A-NK, exhibits poor intracellular accumulation, we argue that lipophilicity is unlikely to lead to different intracellular accumulation properties compared with ketamine and norketamine. Rather, the intracellular accumulation of A-NK suggests that intracellular targets could participate in the unique actions of ketamine^11^,^12^.

The broad intracellular distribution of the analogues (and presumably ketamine/norketamine) also raises the question of specificity of any intracellular actions. Previous reviews of intracellular ketamine actions have suggested that ketamine may have specific intracellular actions^11^,^12^. How could such non-selective distribution of drug be associated with specific, compartmentalized functional effects? One possibility is that not all intracellular binding sites for ketamine and its analogues are functionally relevant^11^.

Norketamine, ketamine, and A-NK each induced strong acute psychotomimetic effects and reduced immobility time in the FST assay. Although limiting compound quantities precluded full time course studies, the FST was performed with a 3 h delay, to help ensure that other acute psychoactive effects did not contribute to FST results. Although NMDAR activity is one commonality among these compounds that could trigger behavioral effects, our data suggest that other intracellular targets might be important for behavioral and perhaps synaptic effects of ketamine analogues, some of which take several hours to manifest in both humans and rodents^6^,^9^,^41^,^42^. This could explain why the pharmacologically similar compound memantine does not share ketamine’s antidepressant-like effects^9^. Memantine, unlike A-NK, ketamine, and norketamine, is nearly fully protonated at physiological pH (pKa 10.3, 99% protonated at physiological pH^20^), which may prevent it from entering cells.

Another possibility is that a common downstream metabolite of the ketamine core structure mediates the antidepressant-like effect^10^. A common metabolite for both ketamine and norketamine is 6-hydroxynorketamine^10^, a neuroactive compound but weak NMDAR antagonist^43^. The cyclohexane ring, which is modified in both ketamine and norketamine to form these hydroxyketamine and hydroxynorketamines, can also be modified in A-NK. Although ketamine is rapidly metabolized to produce norketamine through demethylation, our observed differences as well as those reported previously^17^ suggest that longer alkyl chains such as those in A-NK and A-NK-amide are not lost through metabolic pathways, leaving the terminal alkyne intact. It therefore seems unlikely that a common downstream metabolite of ketamine, norketamine and A-NK can explain the behavioral effects, although it is possible that a common metabolite helps explain the acute behavioral effect of A-NK-amide observed at high concentration (Fig. 2b,c). Furthermore, cellular esterase/amidases are unlikely to explain the lack of cellular labeling or weak behavioral activity of A-NK-amide; precedent examples from the literature on local anesthetics show that amidation is correlated with increased *in vivo* stability (procainamide, lidocaine) over that of procaine^44^,^45^,^46^.

## Methods

### Mice and drug treatments

The study was carried out in strict accordance with the recommendations in the Guide for the Care and Use of Laboratory Animals of the National Institute of Health. The protocol(s) was/were approved by the Washington University Animal Studies committee. All efforts were made to minimize animal discomfort by use of anesthesia/analgesia.

Male C57BL/6 J mice (Jackson Labs) that were 2–2.5 months old were administered ketamine (3–100 mg/kg), norketamine (3–100 mg/kg), A-NK (10–100 mg/kg), A-NK-amide (10–100 mg/kg), or vehicle via intraperitoneal injection. Vehicle was matched across all conditions for each experiment and included up to 30% DMSO or 7% Cremophor-EL in 0.9% saline. Drug effects on behavioral performance were evaluated immediately (acute psychoactivity studies) or three hours (forced swim test, FST) post- injection.

### Forced Swim Test (FST)

Mice were transported from a housing room to the test room where they remained for at least 1 h before testing commenced. The FST procedure involved testing drug- and vehicle-treated mice by placing a mouse inside one of two 2000 ml glass beakers (144 mm diam.) that contained 1600 ml of water at 24 ± 1 °C where each remained for a 6-min trial. An opaque partition was placed between the beakers, and the water was changed for each pair of subjects. Testing of drug- and vehicle-treated mice was counterbalanced across the two beakers with typically one drug- and one vehicle-injected mouse being tested at the same time. A video camera positioned above the beakers was used to record an overhead view of the mice, which was used to evaluate their performance. Immobility, defined as a lack of extraneous movement except for that required to keep the head above water, was quantified (seconds) during the last 4 min of the trial by an observer who viewed the video recording and was unaware of the treatment status of individual mice.

### Locomotor activity

To examine the effects of the drug treatments on general activity/exploratory behavior levels, mice were evaluated over a 1-h period in transparent polystyrene enclosures measuring 47.6 × 25.4 × 20.6 cm high, according to our previously published procedures^47^. Each enclosure was surrounded by two frames containing pairs of photocells. Beam breaks were recorded by a computer, and measured parameters were quantified by standard algorithms (MotorMonitor, Kinder Scientific LLC, Poway, CA). Dependent variables that were analyzed included total ambulations (whole body movements), which were derived from a lower frame used to measure horizontal movement, and vertical rearing frequency, which was computed using a second frame that was raised slightly above the first.

The effect of high doses of ketamine (50–100 mg/kg) and analogues (100 mg/kg) were assessed using the locomotor instrumentation described above and a modified procedure previously utilized with rats to evaluate the effects of high doses of ketamine and metabolites^48^. Relevant dependent variables were ambulations and vertical rearing, which served as measures of psychoactivity (see Results). The acute, high-dose effects of ketamine on these behaviors have been interpreted as psychotomimetic-like effects^18^. Animals dosed with vehicle, ketamine, norketamine, A-NK, and A-NK-amide were all evaluated during the same session, in an interleaved design, immediately following i.p. injection.

The same instrumentation and general procedures described above were used to assess possible activity-related drug effects in the FST studies by evaluating the same doses with the former methods.These control studies involved mice that had served as vehicle controls in the FST. Locomotor assessments were performed 1–2 weeks later on the 1-h locomotor activity test following drug (10 mg/kg) or vehicle injections using the same post-injection delay interval (3 h) that was utilized in the FST studies.

### Hippocampal cultures

Hippocampal cultures were prepared as either mass cultures or microcultures (as indicated in figure legends) from postnatal day 1–3 female rat pups anesthetized with isoflurane, under protocols consistent with NIH guidelines and approved by the Washington University Animal Studies Committee. Methods were adapted from earlier descriptions^49^,^50^,^51^,^52^. Hippocampal slices (500 μm thickness) were digested with 1 mg ml^−1^ papain in oxygenated Leibovitz L-15 medium (Life Technologies, Gaithersburg, MD, USA). Tissue was mechanically triturated in modified Eagle’s medium (Life Technologies) containing 5% horse serum, 5% fetal calf serum, 17 mM D-glucose, 400 μM glutamine, 50 U ml^−1^ penicillin and 50 μg ml^−1^ streptomycin. Cells were seeded in modified Eagle’s medium at a density of ~650 cells mm^−2^ as mass cultures (onto 25 mm cover glasses coated with 5 mg ml^−1^ collagen or 0.1 mg ml^−1^ poly-D-lysine with 1 mg ml^−1^ laminin) or 100 cells mm^−2^ as microcultures (onto 35 mm plastic culture dishes coated with collagen microdroplets on a layer of 0.15% agarose). Cultures were incubated at 37 °C in a humidified chamber with 5%CO_2_/95% air. Cytosine arabinoside (6.7 μM) was added 3–4 days after plating to inhibit glial proliferation. The following day, half of the culture medium was replaced with Neurobasal medium plus B27 supplement (Life Technologies).

### Electrophysiology

Whole-cell recordings were performed at room temperature from neurons cultured for 4–7 days (exogenous applications of agonist) or for 10–12 days (EPSCs) using a Multiclamp 700B amplifier (Molecular Devices, Sunnyvale, CA, USA) and Digidata 1440 A converter with Clampex 10.1 software. Young cells were favored for biophysical experiments with exogenous applications to minimize spatial voltage-clamp errors and to keep current amplitudes small. For recordings, cells were transferred to an extracellular (bath) solution containing (in mM): 138 NaCl, 4 KCl, 2 CaCl_2_, 10 glucose, 0.01 glycine and 10 HEPES, pH 7.25 adjusted with NaOH. Solutions were nominally Mg^2+^ free to promote NMDAR activation. D-(-)−2-Amino-5-phosphonopentanoic acid (D-APV, 25–50 μM) was included until seal formation to prevent excitotoxicity. Solutions with adjusted composition described below were perfused using a gravity-driven local perfusion system from a common tip. The estimated solution exchange times were <100 ms (10–90% rise), estimated from junction current rises at the tip of an open patch pipette. For synaptic recordings, these solutions contained (in mM) 0.001 2,3-dihydroxy-6-nitro-7-sulfonyl-benzo[f]quinoxaline (NBQX) and 0.025 bicuculline methobromide or 0.01 gabazine. EPSCs were elicited from single-cell microcultures by brief depolarization to 0 mV to elicit a breakaway axonal action potential^49^. For exogenous NMDA application, 0.25 mM CaCl_2_ was used (in APV-free perfusion solutions) to minimize Ca^2+^-dependent NMDAR desensitization^53^,^54^. This concentration balanced stability of NMDA responses with cell/membrane seal stability. Tetrodotoxin (250 nM) was added to prevent network activity when evoked synaptic activation was not required. Unless otherwise noted, exogenous NMDA concentration was 30 μM. The open-tip resistance of patch pipettes was 3–6 MΩ when filled with an internal solution containing (in mM): 130 cesium methanesulfonate, 0.5 CaCl_2_, 5 EGTA, 4 NaCl, and 10 HEPES at pH 7.25, adjusted with KOH. Holding voltage was typically −70 mV unless otherwise noted. Access resistance for all recordings was 8–10 MΩ and was compensated for current amplitudes >2 nA.

### HEK Cells and transfection

Procedures were essentially similar to those previously published by our group^55^,^56^. Briefly, HEK 293 cells were cultured in Dulbecco’s modified Eagle’s medium (Life Technologies) supplemented with 10% fetal bovine serum and 1 mM glutamine. Cells on a 35 mm culture plate were transfected with wild-type GluN1a subunit DNA (pRc/CMV vector; 0.3 μg), GluN2B subunit DNA (pcDNA1; 1 μg), and DsRed fluorescent protein DNA (pDsRed2–1;Clontech, Mountain View, CA 0.05 μg). The separate plasmids were co-transfected using Lipofectamine2000 (Life Technologies) according to the manufacturer’s protocol. Using this method, DsRed fluorescence predicts functional NMDAR expression with more than 80% accuracy^55^,^56^.

### Confocal Imaging

For evaluating labeling of A-NK and A-NK-amide, stocks of 10 mM compound were prepared in DMSO and diluted to 10 μM in culture medium for 1 h at 37 °C. The drugs were then washed from the cells three times with PBS. Cells were then fixed with 4% paraformaldehyde and 0.05% glutaraldehyde for 10 min. Cells were washed with PBS and then exposed to click reaction labeling for 1 h in the dark using azide-conjugated Alexa Fluor 488 (1 μM). The click buffers contained 100 μM Tris[(1-benzyl-1*H*-1,2,3-triazol-4-yl)methyl]amine (TBTA), 2 mM (+)- Sodium L-ascorbate in distilled water, 1 μM azide-Alexa Fluor 488 in DMSO, 1 mM CuSO_4_. Pilot experiments demonstrated that additional membrane permeabilization beyond cell fixation was not required for azide-Alexa Fluor 488 entry. Experiments examining *in situ* click labeling compared A-NK and A-NK-amide click labeling with control conditions in which all click reagents were present except pre-incubation in A-NK or A-NK. As expected, the control fluorophore incubation yielded some background fluorescence above that obtained with no fluorophore (46.2 ± 4.0 fluorescence units to 105.2 ± 8.4 fluorescence units from 20 fields in four independent experiments). All reported fluorescence values in text and figures represent labeling above the background labeling performed with all click reagents (including fluorophore) present, but without pre-incubation in unlabeled analogue. For antibody co-labeling experiments, we incubated in 10 μM A-NK for 1 h at 37 °C then fixed cells with 4% paraformaldehyde in phosphate buffered saline for 10 min. After washing, we incubated in primary antibody (anti-PDI, Giantin, or COX IV at 1:2000) in 4% normal goat serum and 0.04% Triton X-100 in phosphate buffered saline for 2 h. Cells were subsequently incubated in secondary antibody (Alexa Fluor 647, 1:500) for 1 h, then clicked (azide-Alexa Fluor 488) for another hour in the above conditions.

### Calculated LogP and pKa derivations

Calculated LogP (cLogP) and pKa values were calculated using software available on the virtual computational chemistry laboratory, www.vcclab.org^57^. cLogP values are represented as the average ± SD of the output by 6 different algorithms consulted for the calculation. pKa values were calculated using SPARC online software^58^ or were obtained from the literature^59^.

### Drugs and reagents

Anti-PDI (Abcam Cat# ab2792, RRID:AB_303304), Anti-COXIV (Abcam Cat# ab16056, RRID:AB_443304) and Anti-Giantin (Covance Research Products Inc Cat# PRB-114C-200, RRID:AB_10063713) antibodies were all chosen for established specificity^60^,^61^,^62^. (R,S)-Ketamine and (R,S)- norketamine were obtained from Tocris as racemic mixtures. Racemic (R,S)-A-NK and (R,S)-A-NK-amide were synthesized as described below, starting from racemic norketamine. Norketamine was synthesized using previously published methods^17^; and the alkyne (A-NK) or amide (A-NK-amide) were synthesized by alkylation or amidation of norketamine. There was no attempt to resolve diasteromers, and all subsequent transformations likely resulted in racemic products. ^1^H NMR and ^13^C NMR spectrometry was performed on an I400 Varian Inova NMR instrument (Agilent, Palo Alto, CA, 400 MHz for ^1^H NMR and 100.5 MHz for ^13^C NMR). High resolution positive ion electrospray mass spectra were obtained on a Bruker MaXis 4 G Q-TOF mass spectrometer.

### Alkyne-Norketamine, A-NK (1)

Norketamine (13 mg, 0.058 mmol) and finely ground potassium carbonate (16 mg, 0.12 mmol) were dissolved in DMF (N, N-dimethylformamide) in a 5-ml reaction vessel. 5-Iodo-1-pentyne (33.8 mg, 0.17 mmol) was added to the solution. The vessel was sealed, and the reaction solution was heated to 100 °C for 24 h. After the reaction solution was cooled to room temperature, the solution was dissolved in dichloromethane (5 ml), and washed with 10% sodium thiosulfate (1 ml × 2), and brine (1 ml × 2). The organic phase was separated and dried over anhydrous sodium sulfate. The solution was filtered under vacuum and concentrated to viscous liquid. The crude product was purified by silica gel column chromatography eluting with hexane: ethyl acetate (3:2) to afford a light yellow viscous liquid (16 mg, 95.2%). ^1^H NMR (400 MHz, CDCl_3_): δ 7.54 (dd, *J* = 7.6 Hz, 1 H), 7.35 (dd, *J* = 8.0 Hz, 1 H), 7.30 (t, *J* = 7.6 Hz, 1 H), 7.22 (t, *J* = 7.6 Hz, 1 H), 2.78–2.74 (m, 1 H), 2.51–2.40 (m, 3 H), 2.23–2.20 (m, 2 H), 2.16–2.13 (m, 1 H), 2.07 (s, 1 H), 2.02–1.98 (m, 1 H), 1.88 (t, *J* = 2.6 Hz, 2 H), 1.80–1.76 (m, 3 H), 1.66–1.61 (m, 2 H). ^13^C NMR (100.5 MHz, CDCl_3_): 209.31, 138.71, 134.03, 131.49, 129.52, 128.92, 126.94, 84.35, 70.15, 68.84, 41.40, 39.76, 39.44, 29.58, 28.22, 22.20, 16.48. HRMS (ESI): calculated for C_17_H_20_ClNO [M+H]^+^: 290.1306; found 290.1302.

### Alkyne-Norketamine Amide, A-NK-amide (2)

4-Pentynoic acid (10.8 mg, 0.11 mmol) and HBTU (43.7 mg, 0.12 mmol) were dissolved in DMF (200 μl) in a 1 ml vial. N, N-diisopropylethylamine (60 μl, 0.34 mmol) was added to the solution, and the reaction solution was stirred for 15 min. Then a solution of norketamine (19 mg, 0.085 mmol) in DMF (65 μl) was added to the solution, and the reaction was stirred at room temperature overnight. The crude product was purified on a short silica gel column with an elution of hexane: ethyl acetate (3:2) to afford a yellow viscous liquid (17 mg, 65.7%). ^1^H NMR (400 MHz, CDCl_3_): δ 7.92 (d, *J* = 8.0 Hz, 1 H), 7.58 (br, 1 H), 7.38–7.22 (m, 3 H), 3.96 (dd, *J* = 14.4 Hz, 1 H), 2.43–2.32 (m, 6 H), 2.08–2.06 (m, 1 H), 1.92 (s, 1 H), 1.83–1.72 (m, 3 H), 1.63 (t, *J* = 14.8 Hz, 1 H). ^13^C NMR (100.5 MHz, CDCl_3_): 209.63, 169.57, 134.74, 133.83, 132.37, 131.15, 129.80, 126.52, 83.03, 69.42, 68.23, 39.45, 38.55, 35.88, 31.24, 22.54, 14.88. HRMS (ESI): calculated for C_17_H_18_ClNO_2_ [M+Na]^+^: 326.0918; found 326.0938.

### Statistical analyses

Behavioral data were analyzed using one-way or repeated measures (rm) analysis of variance (ANOVA) models. These models included one between-subjects variable (Treatment) and the rmANOVAs also included one within-subjects variable (Time Blocks) for analyzing the general ambulatory and vertical rearing data. The Huynh-Feldt adjustment of alpha levels was utilized for all within-subjects effects containing more than two levels to protect against violations of sphericity/compound symmetry assumptions underlying rmANOVA models. Pairwise comparisons were conducted following relevant, significant overall ANOVA effects, which were subjected to Bonferroni correction when appropriate. Upaired *t* tests, Bonferroni corrected for multiple comparisons where appropriate, were used to analyze electrophysiological and cyto-fluorescence results.

## Additional Information

**How to cite this article**: Emnett, C. *et al*. A Clickable Analogue of Ketamine Retains NMDA Receptor Activity, Psychoactivity, and Accumulates in Neurons. *Sci. Rep.* **6**, 38808; doi: 10.1038/srep38808 (2016).

**Publisher's note:** Springer Nature remains neutral with regard to jurisdictional claims in published maps and institutional affiliations.
